# SIRT6’s function in controlling the metabolism of lipids and glucose in diabetic nephropathy

**DOI:** 10.3389/fendo.2023.1244705

**Published:** 2023-10-09

**Authors:** Ying Wang, Tongtong Liu, Yuzi Cai, Weijing Liu, Jing Guo

**Affiliations:** ^1^ Country Renal Research Institution of Beijing University of Chinese Medicine, Key Laboratory of Chinese Internal Medicine of Ministry of Education and Beijing, Dongzhimen Hospital Affiliated to Beijing University of Chinese Medicine, Beijing, China; ^2^ Guang’anmen Hospital, China Academy of Chinese Medical Sciences, Beijing, China; ^3^ Institute of Basic Research in Clinical Medicine, China Academy of Chinese Medical Sciences, Beijing, China

**Keywords:** diabetic nephropathy, SIRT6, glucose metabolism, lipid metabolism, treatment

## Abstract

Diabetic nephropathy (DN) is a complication of diabetes mellitus (DM) and the main cause of excess mortality in patients with type 2 DM. The pathogenesis and progression of DN are closely associated with disorders of glucose and lipid metabolism. As a member of the sirtuin family, SIRT6 has deacetylation, defatty-acylation, and adenosine diphosphate-ribosylation enzyme activities as well as anti-aging and anticancer activities. SIRT6 plays an important role in glucose and lipid metabolism and signaling, especially in DN. SIRT6 improves glucose and lipid metabolism by controlling glycolysis and gluconeogenesis, affecting insulin secretion and transmission and regulating lipid decomposition, transport, and synthesis. Targeting SIRT6 may provide a new therapeutic strategy for DN by improving glucose and lipid metabolism. This review elaborates on the important role of SIRT6 in glucose and lipid metabolism, discusses the potential of SIRT6 as a therapeutic target to improve glucose and lipid metabolism and alleviate DN occurrence and progression of DN, and describes the prospects for future research.

## 1 Introduction

Diabetic nephropathy (DN) is the main microvascular complication of diabetes mellitus (DM) (1). Approximately 30–40% of patients with DM will develop DN, the main cause of end-stage renal disease (2). DN is the main cause of mortality in patients with type 2 DM (T2DM) (3). The all-cause mortality of patients with DM and DN is approximately 30 times that of those without DN, and the vast majority of patients with DN die of cardiovascular disease before end-stage renal disease (4). Multiple risk factors accelerate DN progression, including hypertension, hyperglycemia, obesity, insulin resistance, atherosclerotic dyslipidemia, and familial aggregation (5–9). DN pathogenesis is complex and includes glucose metabolism disorders, changes in fatty acid metabolism, oxidative stress, changes in energy utilization, and mitochondrial dysfunction, which can lead to endothelial dysfunction, glomerular sclerosis, inflammatory cell recruitment, renal tubular fibrosis, and other pathological changes (10, 11). Dyslipidemia and renal ectopic lipid accumulation are associated with kidney disease (especially DN) (12). Almost all renal cell types, from mesangial cells (MCs) to podocytes and proximal tubular epithelial cells (PTECs), can deposit lipids (13). Therefore, glucose and lipid metabolism disorders are important causes of DN onset and progression.

High blood glucose levels and excessive carbohydrate intake can produce toxic effects on cells and tissues through hyperglycemia and carbon stress (14). Hyperglycemia stress including reduction of stress, polyol pathway (15–19), hexosamine pathway (20, 21), protein kinase C (PKC) activation pathway (22, 23), advanced glycation end-product pathway (24–26) and oxidative stress (27–29). Excessive uptake of nutrients (including glucose and lipids) causes carbon overload in cells, resulting in accumulation of a large number of reactive acyl metabolites (including malonyl-coa, succinyl-coa, and acetyl-coa) and ultimately leading to protein modification and dysfunction (30, 31), including through protein acetylation (30, 32) and succinylation (30, 33). Long-term exposure to high concentrations of lipids and lipid derivatives can produce lipotoxicity to cells (34). Long-term elevation of free fatty acid (FFA) levels destroys glucose homeostasis, and exposure to high glucose (HG) causes synergistic glucolipotoxicity (35). Lipotoxicity in DM can aggravate glucotoxicity-induced mitochondrial damage (36). Enhanced fatty acid synthesis and inhibition of fatty acid oxidation are the main causes of renal lipid accumulation (37). Renal lipid deposition induces cell damage by activating oxidative stress, inflammation, fibrosis, and apoptosis pathways (38). Aging is not only a risk factor for the occurrence and development of kidney disease (39), but also leads to adipose tissue dysfunction (40) and decreased glucose tolerance (41), which lead to glucose and lipid metabolism disorders. Therefore, changes in carbohydrate and lipid metabolism as well as kidney aging are associated with the development of chronic kidney disease (42, 43).

**Figure 1:**
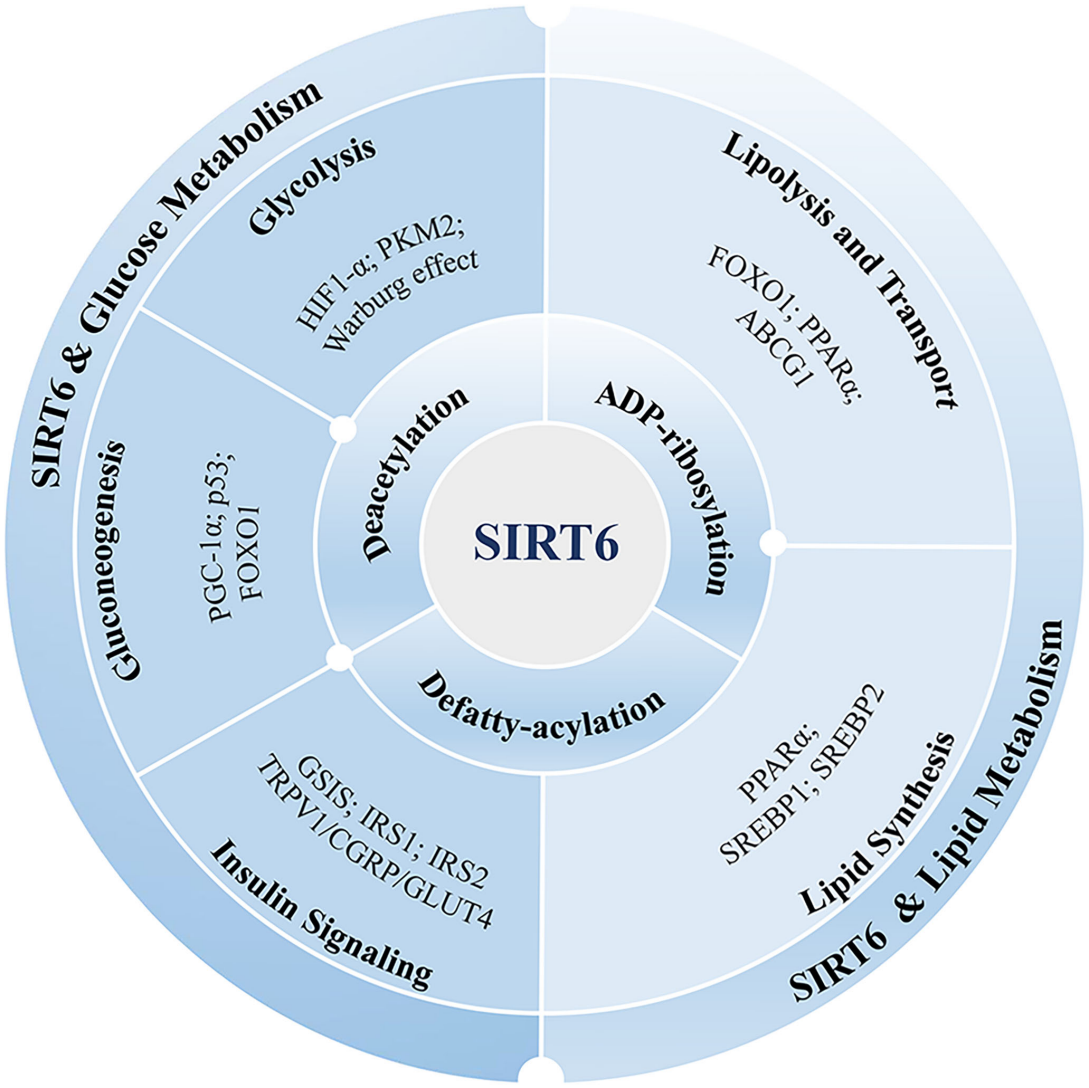
SIRT6’s function in the metabolism of lipids and glucose. SIRT6 exerts its influence on lipid and glucose metabolism through various enzymatic activities, including deacetylation, defatty-acylation, and ADP-ribosylation. These activities enable SIRT6 to modulate metabolic pathways in multiple ways. Glucose metabolism encompasses important processes such as glycolysis, gluconeogenesis, and insulin signaling. SIRT6 is implicated in these processes, and its involvement is associated with several proteins, including HIF-1α, PKM2, PGC-1α, FOXO1, p53, and GLUT4. These proteins play a role in mediating the effects of SIRT6 on glucose metabolism. Lipid metabolism involves the lipolysis, transport, and synthesis of lipids. SIRT6 is involved in regulating lipid metabolism through interactions with various proteins, including FOXO1, PPARα, ABCG1, and SREBP. These proteins collectively contribute to the control of lipid metabolism. By understanding the impact of SIRT6 on these metabolic pathways and its interactions with specific proteins, we can gain valuable insights into its potential as a therapeutic target for managing metabolic disorders.

Sirtuins, as a diverse group of histone deacetylases, that are core participants in anti-aging effects and metabolism (44) and can play an anti-aging role in DN (45, 46). SIRT6 is an important regulator of glucose and lipid metabolism (**Figure 1**) (47–49). It is also involved in anti-aging (45), NAD+ metabolism (50), inflammation (51, 52), autophagy (53, 54) and oxidative stress (55, 56). SIRT6 deacetylase activity prevents the transcription of genes involved in renal fibrosis (57). SIRT6 is a key regulator of DN progression. SIRT6 expression is downregulated in DN kidney tissues (58), and podocyte-specific SIRT6 deletion aggravates podocyte injury and proteinuria in mice with DN (58). In addition, SIRT6 deficiency is associated with mitochondrial and podocyte apoptosis (59, 60).

In this review, we systematically elaborate on the targeting of SIRT6 to regulate glucose and lipid metabolism to delay DN progression and on the feasibility of utilizing SIRT6 in DN treatment.

## 2 Localization, structure, and enzymatic activity of SIRT6

### 2.1 Localization and structure

**Figure 2:**
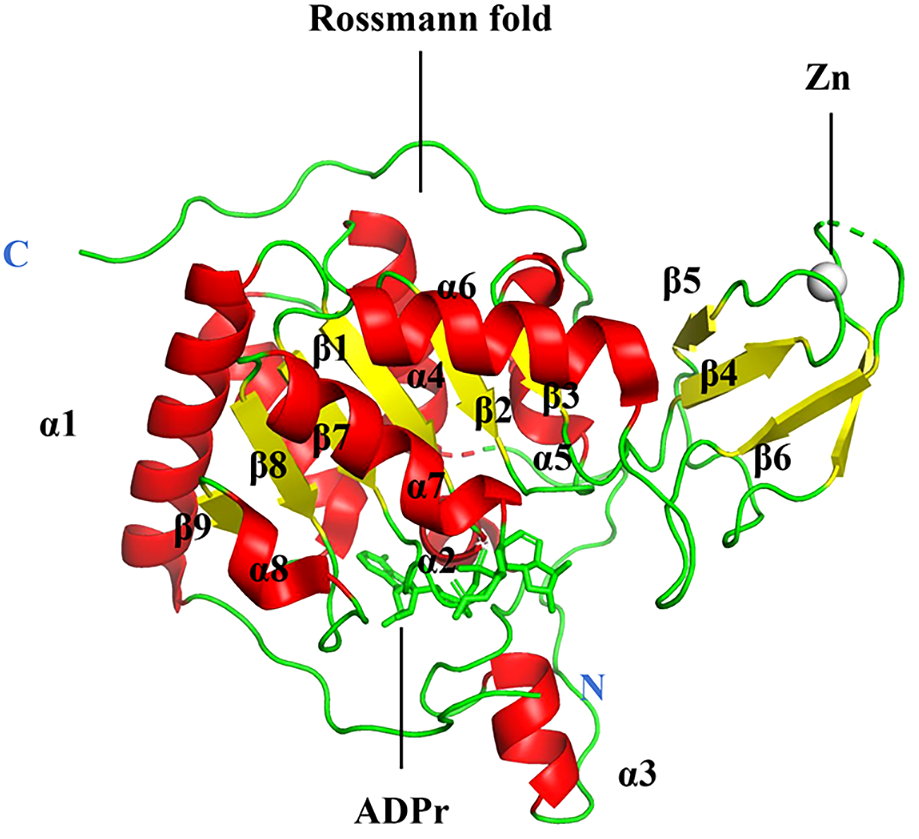
Structure of human SIRT6.

The sirtuin family includes seven proteins (SIRT1–SIRT7), of which SIRT6 is a member of class IV (61). SIRT6 is primarily localized in the nucleus (62). The human *SIRT6* gene contains eight exons, with exon 4 being the shortest at 60 bases and exon 8 the longest at 838 bases (63). The gene is located on chromosome 19p13.3. A protein of 355 amino acids with a projected molecular weight of 39.1 kDa and an isoelectric point of 9.12 is encoded by the human SIRT6 mRNA (63). Most tissues produce SIRT6, and research has shown that its gene is mostly expressed in the embryonic heart, kidney, and brain (64). Eight α-sheets and nine β-strands make up the two globular domains found in SIRT6: a large Rossmann fold for NAD+ binding (residues 25–128 and 191–266) and a smaller zinc-binding domain (residues 129–190). The parallel β-sheets of six strands (β1, β2, β3, β7, β8, and β9) that make up the large Rossmann fold domain are surrounded by two helices (α6 and α7) on one side and four on the other (α1, α4, α5, and α8). The smaller domain, which consists of three antiparallel β-sheets (sheets β4, β5, and β6), is created by two extension loops of the large domain (linking loops β3 and α6) (**Figure 2**) (65). Despite lacking acetylated substrates, SIRT6 has a structurally strong single helix that allows it to bind NAD (65). Deacetylation, defatty-acylation, and ADP-ribosylation are three unique enzymatic activities that SIRT6 has shown (66).

### 2.2 Deacetylation

NAD+-dependent histone deacetylases are the most distinctive features of SIRT6. There are multiple SIRT6-targeted deacetylation sites on histone H3, including at H3K9, H3K18, and H3K27 (67). Histone H3 lysine 9 (H3K9Ac) is the first specific deacetylation substrate that regulates telomere chromatin (68). Lysine 56 in the histone H3 globular nucleus (H3K56Ac) is the second substrate (69) and is involved in DNA repair (70). SIRT6 promotes H3K18 deacetylation in paracentric heterochromatin (71). Further research has shown that the role of SIRT6 as a protein deacetylase extends beyond the scope of histones. C-terminal-binding protein interacting protein (CtIP) was the first discovered non-histone substrate and promotes DNA end resection and homologous recombination (72) to maintain genomic stability. When SIRT6 is activated by ribosomes or fatty acids, its deacetylation activity is significantly enhanced (73, 74).

### 2.3 Defatty-acylation

Fatty acylation of lysine is a novel mechanism that regulates protein secretion (75). Palmitoylation affects cellular protein dynamics and differential regulation (76). Myristoylation affects plasma targeting, subcellular tracking, and protein localization (77). Enzymatic and structural studies have shown that SIRT6 preferentially hydrolyzes long-chain fatty acyl groups (myristoyl and palmitoyl) (73, 78). SIRT6 knockdown increases lysine fatty acylation of the RAS-related protein R-Ras2 (79). SIRT6 regulates the lipid acylation level of K19 and K20 and affects the secretion of tumor necrosis factor α (TNFα) (78). In addition, SIRT6 can remove the fatty acylation of H3K9, H3K18, and H3K27 in fatty-acylated nucleosomes; however, the physiological function of this reaction requires further study (67).

### 2.4 ADP-ribosylation

ADP-ribosylation is a post-translational modification (80) involved in glucose and lipid metabolism (81), DNA repair (82) and cell proliferation (83). SIRT6 is an ADP-ribosyltransferase (62). SIRT6 mono-ADP-ribosylation of KDM2A can locally increase H3K36me2 at DNA damage sites, thereby inhibiting transcription and promoting repair (84). In response to oxidative stress, SIRT6 ribosylates K521 and activates poly (ADP-ribose) polymerase 1 to promote double-strand break repair (85). SIRT6 inhibits long interspersed element 1 retrotransposons by ribosylating KRAB domain-associated protein 1 (86).

### 2.5 Regulation of SIRT6 enzyme activity

Deficiency of SIRT6 SUMOylation specifically reduces H3K56 deacetylation (87). Compared to patients without DM, SIRT6 DNA methylation levels in patients with DM are lower and are negatively correlated with blood glucose levels, suggesting that epigenetic mechanisms regulate SIRT6 expression (88). Oxidative stress inhibited SIRT6 expression in a mouse model of DM embryopathy (89). SIRT6 expression was inhibited by 2,3-dimethoxy-1,4-naphthoquinone (89) *in vitro*. p53 directly activates SIRT6 expression (90, 91). Under normal growth conditions, p53 positively regulates SIRT6 protein levels; however, under nutrient-limited conditions, p53 has no relationship with SIRT6 stability (92). Ubiquitination is a common post-translational modification that regulates target protein stability (93). Ubiquitin-specific peptidase 10 (USP10) inhibits SIRT6 ubiquitination and degradation, reducing liver fat deposition, insulin resistance, and inflammation (94). The ubiquitin ligase CHIP (carboxyl terminus of HSP70-interacting protein) ubiquitinates SIRT6 at K170 (95).

## 3 SIRT6 regulation in glycolipid metabolism

### 3.1 SIRT6 and glucose metabolism

#### 3.1.1 SIRT6 and blood glucose

Cys144 of SIRT6 is a functional redox-sensitive site that regulates glucose metabolism in monocytes (96), including inhibition of glucose transporters and glycolytic enzyme expression (97, 98). SIRT6 inhibitors increase expression of glucose transporters and glycolytic enzymes, reducing blood glucose levels (99). Sirt6-deficient mice exhibit lethal hypoglycemia in early life (100). SIRT6 deficiency did not affect intestinal glucose absorption or renal glucose secretion in mice (98). The kidney regulates glucose homeostasis through gluconeogenesis, glucose uptake from circulation, and glucose reabsorption from glomerular filtrate (101). In DM, the kidneys increase blood glucose by increasing glucose reabsorption in the prourine and upregulating gluconeogenesis in the proximal tubules (PTs) (102). The glucose transporter (GLUT) and sodium-glucose co-transporter (SGLT) are both expressed in renal tissues (103). SIRT6 deletion enhances the membrane association between GLUT1 and GLUT4, thereby enhancing glucose uptake (104). In cell-specific SIRT6 KO mice, SIRT6-mediated forkhead box protein O1 (FOXO1) deacetylation leads to nuclear export and restoration of pancreatic duodenal homeobox 1 (Pdx1) expression. It may also promote glucose-stimulated insulin secretion (GSIS) and upregulate GLUT2 expression (105).

#### 3.1.2 SIRT6 and glycolysis

Glycolysis, a key energy production process in almost all mammalian cells, converts glucose into pyruvate. Under aerobic conditions, it enter the mitochondria (106). When cells are deprived of nutrients or under hypoxia, they undergo anaerobic respiration and convert pyruvate to lactate (107–109). Hyperglycemic toxicity can be reduced by increasing glycolysis. The elevation of enzymes involved in the metabolism of free glucose and its metabolites in glomerular cells is related to the maintenance of renal function in T2DM (110). Anaerobic glycolysis and glucose fermentation into lactate are the main metabolic pathways in podocytes. Under physiological conditions, podocytes do not rely on mitochondrial energy sources, but metabolize glucose to lactate to meet energy demands, similar to the Warburg effect (111). In DN, regulating glucose metabolism, reducing the levels of glucotoxic products, and improving mitochondrial function can protect the kidneys (112). After 8 days of hyperglycemia intervention in renal tubular cells, the downregulation of respiratory parameters persisted and glycolysis increased to compensate (113).

Hypoxia-inducible factor-1α (HIF-1α) regulates glycolytic gene expression. HIF-1 activates glycolytic genes such as pyruvate dehydrogenase kinase (PDK), which is key to hypoxic metabolism adaptations by increasing the conversion rate of glucose to pyruvate and lactate (114). SIRT6 negatively regulates HIF-1α to regulate glycolysis. The two SIRT6 Cys residues Cys18 and HIF-1α (Cys800) form a reversible disulfide bond, thereby inhibiting the transcriptional activity of HIF-1α (115). In a cross-sectional study of patients with T2DM (313 cases), patients with pre-DM (102 cases), and healthy volunteers (100 cases), SIRT6 was elevated in patients with different severities of DM and microalbuminuria with increased TNFα, HIF1-α, and urinary protein biomarkers (116). Thus, HIF1-α is a target for SIRT6 intervention in glycolysis. In SIRT6-deficient cells, HIF-1α protein synthesis and stability are increased, leading to the overexpression of HIF-1α target genes involved in glycolysis, such as those coding for lactate dehydrogenase, triose phosphate isomerase, aldolase, and the rate-limiting glycolytic enzyme phosphofructokinase (98). Under normal nutritional conditions, SIRT6 acts as a histone deacetylase to inhibit the expression of glycolytic genes and maintain an appropriate flux of glucose into the tricarboxylic acid cycle (98). Under nutritional stress, SIRT6 inactivation can activate HIF-1α and recruit p300. Acetylation of H3K9 at the promoter increases the expression of a variety of metabolic genes, resulting in increased glycolysis and decreased mitochondrial respiration (98). In mice specifically overexpressing pyruvate kinase M2 (PKM2) in podocytes, PKM2 protects mitochondrial function in all glomerular cells by activating and inducing the HIF-1α/VEGF pathway, resisting hyperglycemic toxicity, and slowing down DN progression (117). PKM2 activation protects podocytes from glucose-induced injury by increasing glucose metabolic flux, inhibiting the production of toxic glucose metabolites, and inducing mitochondrial biogenesis to restore mitochondrial function (118). SIRT6 deacetylates PKM2, leading to its nuclear export. Therefore, the interaction of SIRT6 with PKM2 and HIF-1α can be further investigated to provide strategies for the treatment of altered podocyte metabolism in DN.

#### 3.1.3 SIRT6 and gluconeogenesis

Gluconeogenesis is an important metabolic process that provides energy to the body, particularly during fasting and physical activities. Systemic SIRT6 overexpression improves the utilization of two major gluconeogenic precursors (glycerol and lactate), blocking age-dependent deterioration of euglycemia and gluconeogenic capacity, indicating that organs other than the liver are critical for SIRT6-mediated gluconeogenesis activation (50). PTs are the second most important gluconeogenic tissue after the liver (119). In DM, both the liver and kidneys increase gluconeogenesis; however, the relative increase in glucose production in the kidneys is much stronger than that in the liver (102). The most important renal gluconeoprecursors are lactate, glutamine, and glycerol (101).

During gluconeogenesis, SIRT6 is regulated by the peroxisome proliferator-activated receptor-γ coactivator 1α (PGC-1α), FOXO1, and other targets. PGC-1α is a key mediator of gluconeogenic gene transcription, and this function depends on its acetylation status (120, 121). Metabolomics suggests that the characteristics of mitochondrial dysfunction in DN are related to decreased expression of the *PGC-1α* gene, which is evidence of the global impairment of mitochondrial biogenesis (122, 123). SIRT6 binds the histone acetyltransferase general control of nucleotide synthesis 5 (GCN5) at K549 to deacetylate it, changing protein phosphorylation to activate GCN5. This in turn suppresses hepatic gluconeogenesis by increasing PGC-1α acetylation (120). p53 downregulates the rate-limiting enzymes of gluconeogenesis (phosphoenolpyruvate carboxykinase 1 and glucose-6-phosphatase) and activates SIRT6 expression. SIRT6 deacetylates FOXO1 and exports it to the cytoplasm to regulate gluconeogenesis (90). SIRT6 also regulates FOXO1 nuclear translocation, affecting renal glucose reabsorption and gluconeogenesis in type 1 DM (124).

#### 3.1.4 SIRT6 and insulin signaling

Insulin is the only hormone in the body that lowers blood glucose levels, and it is secreted by pancreatic β-cells. The kidney plays a major role in insulin degradation, removing 6–8 U of insulin daily via two major pathways (125). The GSIS of pancreatic β-cell SIRT6-knockout mice decreased by approximately 50%, suggesting that SIRT6 activation may improve insulin secretion in DM (126). SIRT6 deficiency also leads to abnormal upregulation of thioredoxin-interacting protein in islet β-cells, thereby inhibiting insulin secretion (127). SIRT6 overexpression can reduce palmitate (PA)-induced lipotoxicity, improve pancreatic β-cell viability, and increase GSIS (128). SIRT6 also regulates GSIS via mitochondrial glucose oxidation, plasma membrane depolarization, and calcium dynamics (126). Furthermore, SIRT6 inhibits multiple upstream molecules, such as insulin receptor, insulin receptor substrate 1, and insulin receptor substrate 2. Additionally, SIRT6 negatively regulates AKT phosphorylation (104).

Insulin resistance (IR) is a factor that promotes DN progression (129). SIRT6 overexpression activates transient receptor potential vallinoid 1 (TRPV1)/calcitonin gene-related peptide (CGRP) signaling and regulates GLUT expression at the protein and mRNA levels, which are involved in the TRPV1-CGRP-GLUT4 signaling axis, thereby increasing glucose intake and reducing IR in mice fed high-fat diets (HFDs) and 3T3-L1 adipocytes (130). Therefore, SIRT6 not only affects insulin secretion and sensitivity, but also serves as a potential target for the treatment of IR.

### 3.2 SIRT6 and lipid metabolism

#### 3.2.1 SIRT6 and adipocytes

Adipose tissue is mainly composed of adipocytes, interstitial fibroblasts, and progenitor cells, which form energy storage organelles in the form of triglycerides packaged into lipid droplets (LDs). Adipose tissue also plays an important role in regulating systemic metabolic homeostasis (131–134). Depending on adipocyte type, fat can be classified as white, brown, or beige. White adipocytes have unilocular LDs mainly responsible for energy storage (131). Brown fat cells are rich in mitochondria that consume energy to produce heat (135). Mice with SIRT6-deficient adipose tissue have shown elevated blood glucose levels and severe IR (136). Obesity, hyperglycemia, and other factors can reduce SIRT6 expression. SIRT6 expression was observed to have decreased in the adipose tissue of db/db mice in a model of T2DM (120). SIRT6 expression in the abdominal adipose tissue of patients with obesity and pre-DM is lower than that in healthy patients, while nuclear transcription factor-κB (NF-κB), peroxisome proliferator-activated receptor γ (PPARγ), and sterol regulatory element-binding protein 1 (SREBP-1) expression levels increase, suggesting their involvement in the inflammatory pathway (137). SIRT6 expression in subcutaneous adipose tissue increases significantly after weight loss (138). Low temperature can induce SIRT6 to interact with the *PGC-1α* promoter and promote phospho-activating transcription factor 2 (p-ATF2) binding, thereby activating thermogenic genes and promoting fat thermogenesis (136). SIRT6 also inhibits preadipocyte differentiation by activating the adenosine monophosphate-activated protein kinase-α (AMPKα) pathway (139). Adipose tissue can also function as a secretory organ for leptin and adiponectin (140, 141). SIRT6 deficiency impairs leptin-induced signal transduction (142). Increased adiponectin can reduce proteinuria, glomerular hypertrophy, and inflammatory responses in the renal tissue (143).

#### 3.2.2 SIRT6, lipolysis, and transport

SIRT6 overexpression significantly reduces blood triglycerides in mice (144). FOXO1 is involved in lipid metabolism, promotes lipolysis, and inhibits adipocyte differentiation. Acetylation and deacetylation are the most important regulatory mechanisms affecting FOXO1 expression and activity (145). SIRT6 is a FOXO1 deacetylase that drives lipid catabolism, and its activity is enhanced by the loss of mTOR complex 2 (mTORC2) (146). mTORC2 promotes glucose uptake and adipogenesis in adipocytes, and counteracts the inflammatory response of macrophages (147). It also regulates lipid metabolism in brown adipocytes via the SIRT6-FOXO1 pathway (146). However, the lack of SIRT6 can increase FOXO1 acetylation, promote FOXO1 nuclear export, and reduce the positive regulation of adipose triglyceride lipase, a key enzyme in fat mobilization (148). PPARα, one of the PPAR isoforms, is a key transcription factor involved in hepatic oxidation. PPARα activates PDK4 to inhibit the oxidation of pyruvate produced by glycolysis and increase the production of lactate and alanine, thereby indirectly promoting lipid oxidation in the liver (149). SIRT6 can bind PPARα and its response elements in the promoter region to activate gene transcription and promote lipid β-oxidation (150). Lipoproteins include phospholipids, free cholesterol, and apolipoproteins (151). Disorders of cholesterol metabolism are also associated with lipotoxicity and lipid accumulation in DM (152). SIRT6 affects cholesterol efflux in podocytes by regulating the expression of ATP-binding cassette transporter G1 (ABCG1) expression. SIRT6 deficiency exacerbates Ang II-induced cholesterol accumulation and podocyte injury SIRT6 is a potential target for renin-angiotensin system-related podocyte injury (153).

#### 3.2.3 SIRT6 and lipid synthesis

SREBP is a lipogenic transcription factor regulated by cholesterol, insulin, and glucose. PPARα can inhibit the SREBP-mediated synthesis of cholesterol and triglycerides (154, 155). SREBP1 regulates adipogenesis by activating the genes involved in fatty acid and triglyceride biosynthesis, whereas SREBP2 activates the genes involved in cholesterol synthesis (156). SREBP1 overexpression in the kidneys induces glomerulosclerosis (157). SIRT6 can bind to the promoter regions of *SREBP1c* and SREBP2 and repress transcription by deacetylating histone H3K56 in the promoter. FOXO3 recruits SIRT6 to the SREBP-2 gene promoter, and SIRT6 deacetylates H3K9AC and H3K56AC to reduce low-density lipoprotein (LDL) cholesterol (158). SIRT6 also inhibits SREBP1c by increasing the adenosine monophosphate (AMP)/ATP ratio and stimulating AMPK phosphorylation (159). miRNAs are key regulators of lipid synthesis, fatty acid oxidation, and lipoprotein formation and secretion (160). However, miR33a and miR33b from the SREBP2 and SREBP1 introns can inhibit SIRT6 expression (159, 161). SIRT6 inhibits lipid deposition by activating the AMPKα pathway (139). Ectopic lipid deposition (ELD) is associated with DN progression (12). SIRT6 improves lipid accumulation via FOXO1 and PPARγ (162). Therefore, SIRT6 can affect the lipogenic transcription factors SREBP1 and SREBP2 through a variety of mechanisms. Further studies are needed to determine whether SIRT6 alleviates renal ELD.

## 4 Effect of glucose and lipid metabolism on DN

**Figure 3:**
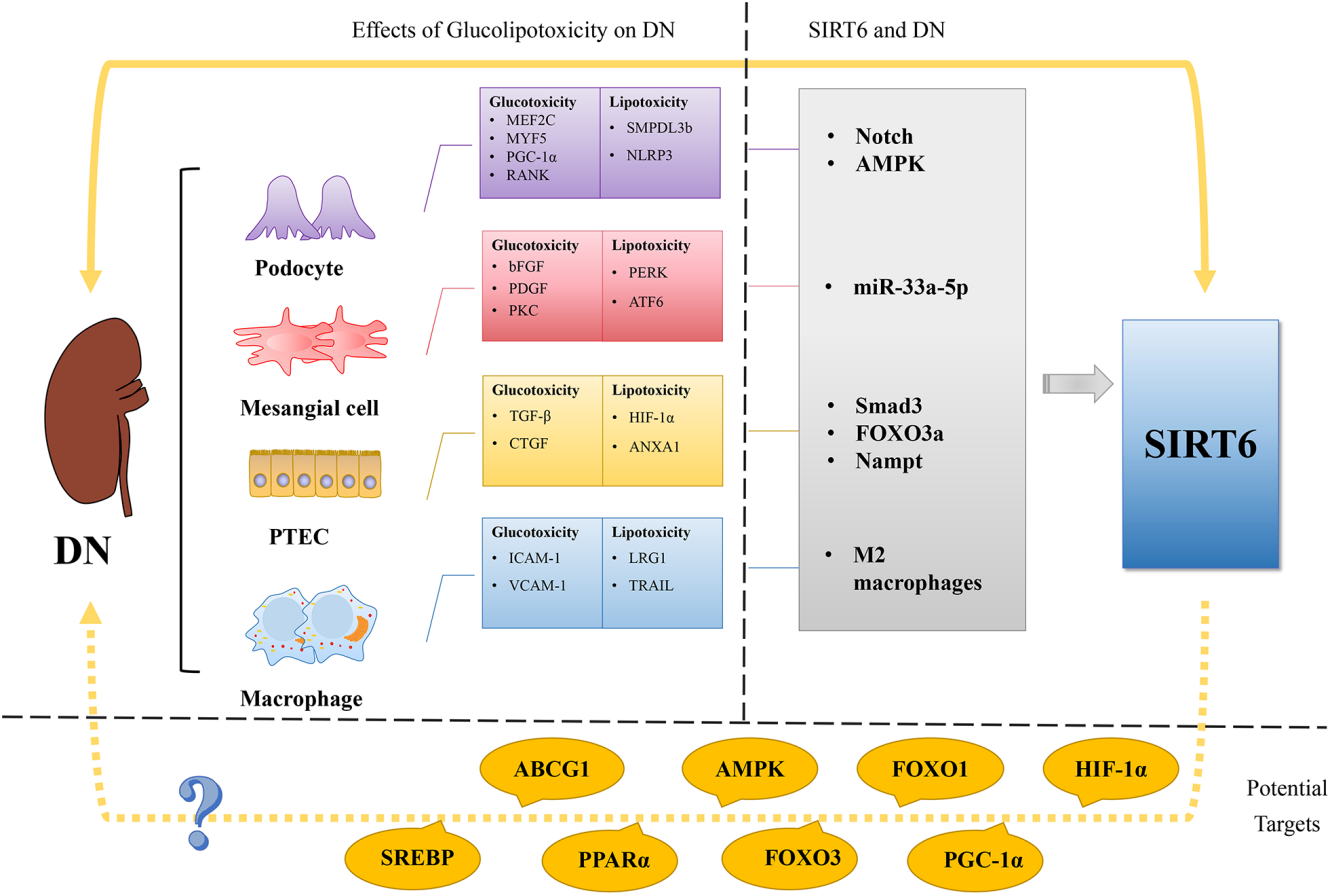
Regulation of glucose and lipid metabolism in DN and SIRT6’s possible role as a treatment target for DN. The imbalance of lipid and glucose metabolism is a crucial etiological component in the development of DN, a microvascular complication of DM. In podocyte glucotoxicity, MEF2C, MYF5, PGC-1α, and RANK are involved, whereas in podocyte lipotoxicity, SMPDL3b and NLRP3 are. The glucotoxicity of mesangial cells is mediated by bFGF, PDGF, and PKC, whereas the lipotoxicity of mesangial cells is mediated by PERK and ATF6. TGF-β and CTGF support glucotoxicity in PTCs, whereas HIF-1α and ANXA1 support lipotoxicity. In contrast to lipotoxicity, which is mediated by LRG1 and TRAIL, macrophage glucotoxicity is mediated by ICAM-1 and VCAM-1. Recent studies show that SIRT6 plays a role in the activation of the Notch pathway, AMPK, miR-33a-5p, Smad3, FOXO3a, Nampt, and M2 macrophages in DN. Potential targets for treating DN using SIRT6 are suggested, including HIF-1α, PGC-1α, FOXO1, FOXO3, AMPK, PPARα, ABCG1, and SREBP, given the involvement of SIRT6 in glucose and lipid metabolism. By targeting SIRT6 and its associated pathways, there is potential to modulate glucose and lipid metabolism and mitigate the development and progression of DN. Further research and investigation are warranted to explore the therapeutic implications of targeting SIRT6 in DN treatment.

Disorders of glucose and lipid metabolism are closely related to the occurrence and progression of DN (**Figure 3**). Glucotoxicity and lipotoxicity can affect a variety of intrinsic renal cells in DN, causing structural and functional changes in the glomeruli and tubules.

### 4.1 Glucotoxicity and DN

DN is a microvascular disease in which vascular endothelial cells are unable to downregulate glucose transport in response to high glucose levels, resulting in a large flow of intracellular glucose that triggers the production of pathogenic mediators (163). Hyperglycemia is considered a key initiating factor in DN-related renal injury. Excess glucose flux generates reactive oxygen species via several pathways (164). Mesangial expansion and podocyte loss are important early features of DN, and tubulointerstitial injury and fibrosis are key to the progression of DN to renal failure (11).

Podocyte structure and dysfunction are the core factors in DN pathogenesis. Hyperglycemia can induce podocytopathy, which is characterized by cell hypertrophy, foot process loss, and podocyte depletion (165, 166). Expression levels of myocyte-specific enhancer factor 2C (MEF2C), myogenic factor 5 (MYF5), and PGC-1α are decreased in renal tissues of patients with DN. This suggests that hyperglycemia reshapes energy metabolism in human podocytes (167). SIRT6 promotes the expression of PGC-1α (168). Receptor activator of NF-κB (RANK) is induced in DM and promotes glomerular oxidative stress as well as the secretion of pro-inflammatory cytokines, leading to podocyte injury and mediating the occurrence of DN (169). SIRT6 attenuates NF-κB signaling via deacetylation of H3K9 on chromatin (170).

Increased glucose load in PTs in the early stage of DM leads to maladaptive hypertrophy, hyperplasia of cortical tubules (171), and, at the same time, upregulated glucose transport (172), which promotes glucose reabsorption. In PTECs, SGLT2 and SGLT1 actively reabsorb glucose and passively return it to the blood via GLUT2 (173). These conditions can activate tubule-glomerular feedback, leading to increased intraglomerular pressure and ultrafiltration (174, 175). SIRT6 regulates the expression of GLUT2 during glucose reabsorption and gluconeogenesis (124). Renal tubular epithelial-to-mesenchymal transition (EMT) and tubulointerstitial fibrosis are important pathological features of DN (176, 177) and represent the “final common pathway” of associated renal function loss (178). Increased extracellular matrix (ECM) deposition in the kidney can be regulated by transforming growth factor-beta (TGF-β), connective tissue growth factor (CTGF), and other profibrotic mediators (179). Human PTECs and cortical fibroblasts exposed to HG show altered cell growth and collagen synthesis independent of hemodynamics and glomerular or vascular pathology (180). SIRT6 attenuates TGF-β-induced fibrosis in renal tubular cells by blocking β-catenin expression (57, 181).

MCs proliferate in the early stages of DN and are closely related to basic fibroblast growth factor (bFGF) and platelet-derived growth factor (PDGF) (182). Studies shows that SIRT6 regulate the expression of PDGF (183). PKC activation by glucose increases the permeability of endothelial cells to albumin, stimulates the synthesis of matrix proteins in MCs, and changes the function and structure of DM glomeruli (184). PKC also phosphorylates SIRT6 to mediate fatty acid β-oxidation (185).

Macrophages are the main immune cells, and activation of resident and infiltrating macrophages in DN can promote inflammation and fibrosis of the glomeruli and tubulointerstitium (186). HG induces high expression of intracellular adhesion molecule-1 (ICAM-1) and vascular cell adhesion molecule-1 (VCAM-1) in vascular endothelial cells, which promotes the recruitment of renal macrophages in DN (187). Deacetylation of MRTF-A by SIRT6 leads to nuclear expulsion, thereby inhibiting the binding of MRTF-A to the ICAM-1 promoter and subsequently inhibiting the transcription of ICAM-1 (188). SIRT6 inhibits monocyte adhesion through downregulation of endothelial VCAM-1 expression (189).

### 4.2 Lipotoxicity and DN

Renal lipotoxicity, caused by lipid metabolism disorders, is involved in DN progression and renal dysfunction. Lipid metabolism disorders are significantly correlated with inflammation, podocyte dysfunction, fibrosis, and estimated glomerular filtration rate (eGFR), while lipid deposition is related to disorders of lipid metabolism genes (152). DM often coexists with obesity and leads to renal lipid accumulation (190). The degree of renal lipid deposition is related to renal function in DN (152, 191). Cholesterol accumulation in podocytes is associated with glomerulosclerosis progression (192). In DN, accumulation of lipids exceeding LD storage damages podocytes and renal tubular cells (193). Compared to healthy patients, patients with obesity have increased phospholipid accumulation, larger lysosomes, and impaired autophagic flux in the kidney (194). HFDs induce autophagolysosome dysfunction in mice accompanied by impaired autophagy, increased hypertrophy, lipid peroxidation and aging markers in the S2 segment of PTECs, sparse peritubular capillaries with localized interstitial fibrosis, and glomerular hypertrophy with mesangial expansion (195).

The expression of sphingomyelinase-like phosphodiesterase 3b (SMPDL3b) is increased in DN podocytes, and SMPDL3b promotes degradation of ceramide-1-phosphate (C1P) to ceramides and sphingolipids, which causes the insulin receptor to shift from the caveolin-1-rich domain in a C1P-dependent manner, leading to impaired AKT phosphorylation and podocyte injury (196, 197). Inhibition of nucleotide-binding oligomerization domain-like receptor protein 3 (NLRP3) inflammasome activation inhibits lipid accumulation and improves podocyte injury (198). SIRT6 is involved in the NLRP3-mediated cell pyroptosis (199).

Healthy PTECs are rich in mitochondria and mainly depend on fatty acid beta-oxidation (FAO) for energy. They activate PGC-1α transcription through multiple signaling pathways, including the mTOR and AMPK pathways. The balance between mitochondrial dynamics and energetics maintains mitochondrial homeostasis (119). A shift from fatty acid utilization to glycolysis and lipid accumulation is a metabolic change characteristic of PTs in the development of DN and progression of renal fibrosis and is associated with increased HIF-1α expression (200). LDs are energy storage cellular organelles closely related to mitochondria (201). A single phospholipid bilayer can isolate neutral lipids from the cytoplasm and protect cells from FFA toxicity (202). Lipophagy occurs when LDs are isolated by autophagosomes and fuse with lysosomes to form autolysosomes, which are subsequently degraded by lysosomal hydrolases within the autolysosomes. This hydrolysis produces FFAs, which are recycled back into the cytoplasm for mitochondrial oxidation (203, 204). Lipophagy deficiency plays a key role in the development of ELD and lipid-related renal injury in DN (205). Overexpression of SIRT6 enhances autophagy (206). Annexin A1 (ANXA1) may improve mitochondrial FAO in PTECs through the AMPK/PPARα/CPT1b signaling pathway, thereby reducing intracellular lipid accumulation and improving lipotoxicity-mediated, DN-related tubular damage (191). The AMPK-SIRT6 pathway is involved in aging-related lipid deposition due to metabolic disorders (207).

MCs are susceptible to lipotoxicity, and lipotoxicity-induced MC apoptosis is related to decreased renal function (208, 209). Lipotoxicity is mediated by protein kinase R-like endoplasmic reticulum kinase (PERK) and activating transcription factor 6 (ATF6) signaling pathway-induced apoptosis in MCs (210). It is found that upregulation of SIRT6 expression inhibited the expression of p-PERK and ATF6 (211, 212).

Macrophages infiltration around apoptotic tubular epithelial cells induced by lipotoxicity has been observed in DN and is associated with leucine-rich α-2-glycoprotein 1 (LRG1) and tumor necrosis factor-related apoptosis-inducing ligand (TRAIL) (213).

## 5 SIRT6 and DN

SIRT6 expression was significantly decreased in HG-stimulated podocytes in a concentration- and time-dependent manner (47, 58). SIRT6 mRNA levels correlate positively with eGFR and negatively with proteinuria in renal biopsies of patients with podocyte disease (58). In streptozotocin (STZ)- and adriamycin (ADR)-treated mice and in db/db mice, SIRT6 expression in the kidneys decreased (58). The reduced expression of SIRT6 in podocytes suggests that SIRT6 reduction is an important cause of podocyte injury under various pathological conditions (58). SIRT6 inhibits the Notch pathway in HG to increase autophagic flux, reduce pro-inflammatory mediators, improve actin cytoskeleton disorders, and attenuate podocyte apoptosis to protect podocytes (58). SIRT6 activates AMPK and inhibits HG-induced mitochondrial dysfunction and podocyte apoptosis (47).

SIRT6 promoted Smad3 deacetylation and inhibits Smad3 nuclear accumulation to alleviate DN kidney injury in HG-induced HK-2 cells and in db/db mice. FOXO3a binds to the SIRT6 promoter and enhances its expression to prevent EMT and renal tubular injury in DN and can mediate SIRT6/Smad3 signaling to treat DN (214). Albuminuria decreases nicotinamide phosphoribosyltransferase (Nampt) expression in the PTs of STZ-induced diabetic mice, ultimately leading to matrix metalloproteinase (MMP) inactivation and reduced fibrous tissue disintegration by increasing H3K9 acetylation and decreasing SIRT6 expression in the tissue inhibitor of metal protease 1 (TIMP-1) promoter. Therefore, ECM remodeling linked to DN fibrosis can be efficiently controlled by the Nampt–SIRT6 axis within PTs (215).

In HG-induced rat MCs and STZ-induced DM mice, circ-ITCH regulated SIRT6 expression through miR-33a-5p to reduce inflammation and fibrosis (216).

In addition, SIRT6 protected podocytes from injury in a simulated DN microenvironment by activating M2 macrophages (217). Although there have been few reports on the relationship between SIRT6 and glomerular endothelial cells, SIRT6 has been shown to protect endothelial cells and exert anti-atherosclerotic effects. SIRT6 attenuates the endothelial dysfunction induced by cholesterol crystals by activating nuclear erythroid 2-related factor 2 (Nrf2) (218). SIRT6 deacetylates and reduces the expression of tumor necrosis factor ligand superfamily member 4 (TNFSF4) to maintain endothelial cell function and mitigate atherosclerosis (189).

## 6 Potential therapies targeting SIRT6 in DN

Recently, SIRT6 has been shown to play therapeutic roles in various diseases. Small molecules and compounds that regulate SIRT6 include MDL-811 (219) in ischemic brain injury, UBCS039 (220, 221) in cancer and liver injury, and anthocyanins in osteoarthritis (222). Because SIRT6 can inhibit glycolysis, it is considered part of a potential new generation of anticancer treatment targets (223).

Drugs targeting SIRT6 also play important roles in the treatment of DN (**Table 1**). Diosgenin can reduce lipid accumulation by regulating SIRT6 in early DN while affecting PDK4 and angiopoietin-like-4 (ANGPTL4) to protect podocytes and reduce damage (224). After ginsenoside Rb3 treatment of palmitic acid-induced podocytes (CIHP-1 cells), PPARδ and SIRT6 expression increased in a dose-dependent manner and reduced inflammation and oxidative stress, thereby reducing podocyte apoptosis (225). Yishen Tongluo formula (YSTLF) treatment of db/db mice improved renal injury and fibrosis by positively regulating SIRT6 expression, inhibiting the TGF-β1/Smad2/3 signaling pathway and promoting TGF-β1 degradation (226). IR is closely associated with DN (228). Total sesquiterpene glycosides in loquat leaves can promote the SIRT6/Nrf2 signaling pathway to improve IR (227).

**Table 1:** Drugs targeting SIRT6 for renal glucose and lipid metabolism.

Drugs	Targets	Biologic effects	Experimental models	References
Diosgenin	SIRT6; PDK4; ANGPTL4	reduced lipid accumulation; protected against podocyte injury	DN model (db/db mice)	(224)
Ginsenoside Rb3	PPARδ; SIRT6	alleviated inflammation; alleviated oxidative stress; attenuated podocytes apoptosis	hyperlipidemia (CIHP-1 cells)	(225)
Yishen Tongluo formula	Sirt6/TGF-β1/Smad2/3 pathway	promoted degradation of TGF-β1; ameliorated renal damages and fibrosis	DKD model (db/db mice) (SV40-MES-13 cells)	(226)
Loquat leaves total sesquiterpene glycosides	IRS-1/GLUT4 pathway; AMPK; TRPV1; SIRT6/Nrf2 pathway	ameliorated IR; anti-inflammatory and antioxidant; improved glucose and lipid metabolism	insulin resistance (C57BL/6 mice)	(227)

## 7 Conclusions and perspectives

Accumulating evidence suggests that SIRT6 plays a key role in DN treatment. This review describes the structure and enzymatic activity of SIRT6 and summarizes its important role in glucose and lipid metabolism. Additionally, we described the regulation of glucose and lipid metabolic pathways, including glycolysis, gluconeogenesis, lipolysis, and lipid synthesis, achievable by targeting SIRT6 to affect the progression of DN. Several compounds act as SIRT6 agonists and play potential roles in the treatment of DN. We have focused on the role of the sirtuin family in kidney diseases, especially in DN (46, 162, 229). However, the role of SIRT6 in regulating glucose and lipid metabolism remains unclear. As a potentially underappreciated and understudied target in DN, many challenges remain in the study of SIRT6. Most studies on SIRT6 have been preclinical, and the focus should be shifted to clinical applications, as well as to the efficacy, safety, and stability of targeted drugs. In conclusion, further exploration of the properties of SIRT6 is of potential value, and targeting SIRT6 has important clinical implications for the treatment of DN.

## Author contributions

YW and JG conceived and designed the study. YW and TL wrote the manuscript. YW and TL designed the figures and edited the manuscript. JG and YC revised the paper. WL and JG supervised the writing. All authors have read and approved the final version of manuscript. All authors contributed to the article and approved the submitted version.
